# Neuropeptide Alterations in Type 2 Diabetes Mellitus: A Meta‐Analysis of Circulating Spexin and Galanin Levels

**DOI:** 10.1002/edm2.70303

**Published:** 2026-08-10

**Authors:** Roshan Kumar Mahat, Sushil Yadav, Kahkashan Naaz, Nand Kishor Prasad Sah, Amrendra P. Kushwaha

**Affiliations:** ^1^ Department of Biochemistry, Teerthanker Mahaveer Medical College & Research Centre Teerthanker Mahaveer University Moradabad India; ^2^ Department of Biochemistry Shri Balaji Institute of Medical Sciences Raipur India; ^3^ Department of Physiotherapy Teerthanker Mahaveer University Moradabad India; ^4^ Nepal Medical College and Teaching Hospital Kathmandu Nepal

**Keywords:** galanin, meta‐analysis, neuropeptides, Spexin, type 2 diabetes mellitus

## Abstract

**Background:**

Spexin and galanin are neuropeptides involved in glucose homeostasis, insulin sensitivity, appetite, and energy metabolism. We conducted a systematic review and meta‐analysis to synthesise evidence on circulating spexin and galanin concentrations in patients with type 2 diabetes mellitus (T2DM).

**Methods:**

PubMed/MEDLINE, Scopus, and Web of Science were systematically searched from inception to March 22, 2026. Observational studies reporting circulating spexin and/or galanin levels in adults with T2DM and non‐diabetic controls were included. Random‐effects meta‐analyses were performed to calculate pooled standardised mean differences (SMDs) with 95% confidence intervals (CIs). Subgroup analyses, meta‐regression, sensitivity analyses, prediction intervals, and publication bias assessments were conducted.

**Results:**

Nineteen studies met the inclusion criteria. Thirteen studies (1453 T2DM and 850 controls) showed significantly lower circulating spexin levels in T2DM (SMD = −2.37, 95% CI: −3.21 to −1.53; *p* < 0.0001), with substantial heterogeneity (I^2^ = 98.3%). Four studies further showed reduced spexin levels in patients with diabetic complications compared with uncomplicated T2DM (SMD = −1.12, 95% CI: −1.75 to −0.49; *p* = 0.0005). Meta‐regression identified age as a significant moderator (*p* = 0.007), whereas BMI, HbA1c, sex, and publication year were not significant. Four studies (171 T2DM and 172 controls) revealed significantly elevated circulating galanin levels in T2DM (SMD = 1.35, 95% CI: 1.02–1.68; *p* < 0.0001) with moderate heterogeneity (I^2^ = 47.2%).

**Conclusions:**

Circulating spexin levels are significantly decreased, whereas galanin levels are significantly increased in T2DM. These neuropeptides show potential as biomarkers of metabolic dysregulation and diabetic complications; however, the findings should be interpreted with caution owing to the very low certainty of the available evidence.

## Graphical abstract

This systematic review and meta‐analysis demonstrates that circulating spexin levels are significantly reduced, whereas galanin levels are significantly elevated, in patients with type 2 diabetes mellitus. Despite their potential as biomarkers of metabolic dysregulation, the current evidence remains of very low certainty.

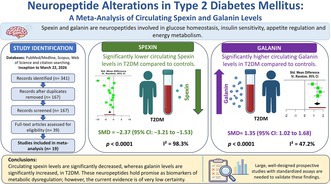

## 1 Introduction

Type 2 Diabetes Mellitus (T2DM) is a multifactorial endocrine and metabolic disease marked by persistent elevation of blood glucose levels resulting from defective insulin action and progressive pancreatic β‐cell dysfunction. Over recent decades, T2DM has emerged as a major public health concern due to its rapidly increasing prevalence, long‐term complications, and considerable socioeconomic impact on healthcare systems worldwide. The development of T2DM is influenced by a complex interaction between inherited susceptibility and modifiable lifestyle factors such as obesity, unhealthy dietary habits, physical inactivity, and sedentary behaviour [1].

According to the International Diabetes Federation (IDF) Diabetes Atlas, an estimated 589 million adults were living with diabetes worldwide in 2024, and this number is projected to increase to 853 million by 2050. Approximately 90% of these cases are T2DM. Diabetes was responsible for an estimated 3.4 million deaths globally in 2024, while its associated healthcare expenditure exceeded USD 1 trillion, highlighting its enormous clinical and economic impact [2]. Furthermore, chronic hyperglycemia predisposes affected individuals to debilitating microvascular complications, including diabetic nephropathy, retinopathy, and peripheral neuropathy, as well as macrovascular complications such as coronary artery disease, stroke, and peripheral arterial disease, which collectively contribute substantially to disability, premature mortality, and reduced quality of life [2, 3]. Consequently, the identification of reliable biomarkers that facilitate early diagnosis, risk stratification, and monitoring of disease progression remains an important research priority.

Recent studies have highlighted the important role of neuropeptides and peptide hormones in the regulation of glucose metabolism, lipid homeostasis, insulin secretion, appetite control, and energy balance. Among these peptides, Spexin (SPX) and Galanin (GAL) have gained increasing attention because of their potential involvement in metabolic disorders, including obesity and T2DM. Both SPX and GAL are widely expressed in the central nervous system as well as peripheral tissues in humans and other vertebrate species. Their biological actions are mediated through galanin receptors (GALRs), namely GALR1, GALR2, and GALR3. Galanin is capable of binding to all three receptor subtypes, whereas spexin predominantly interacts with GALR2 and GALR3, indicating partially overlapping yet distinct physiological functions [4].

Spexin is a recently identified 14–amino acid peptide discovered through bioinformatics analysis of the human proteome. It is expressed in multiple tissues, including the hypothalamus, liver, kidney, pancreas, thyroid gland, adipose tissue, and gastrointestinal tract, suggesting a broad physiological role in endocrine and metabolic regulation. Previous evidence has demonstrated that spexin participates in glucose metabolism, insulin sensitivity, appetite regulation, and lipid homeostasis [5]. Experimental studies indicate that exogenous SPX administration promoted weight loss, stimulated *β* cell proliferation and glucose‐stimulated insulin secretion (GSIS), improved glycemic control, and suppressed hepatic glucose production [6, 7]. Additionally, several clinical investigations have reported reduced circulating spexin levels in individuals with obesity and T2DM, supporting its possible role in metabolic dysfunction and insulin resistance [8, 9]. Emerging evidence further suggests that decreased spexin levels may also be associated with the development and progression of diabetic complications, including diabetic nephropathy [10], diabetic peripheral neuropathy [11], diabetic retinopathy [12], and cardiovascular disease [13], indicating a potential role of spexin in the pathophysiology of both metabolic dysregulation and chronic diabetes‐related complications.

Galanin is another important neuropeptide implicated in the regulation of glucose homeostasis, appetite, energy expenditure, and insulin sensitivity. Initially isolated from porcine intestine, galanin exerts its physiological effects through three receptor subtypes: GALR1, GALR2, and GALR3 [4]. Galanin and spexin share an evolutionary relationship and partially overlapping receptor signalling pathways, suggesting a possible interaction in metabolic regulation. GAL and related peptides, including galanin‐like peptide (GALP) and galanin message‐associated protein (GMAP), are widely distributed in the hypothalamus and peripheral tissues involved in metabolic control [14, 15]. Experimental studies indicate that galanin promotes glucose transporter‐4 (GLUT4) translocation in insulin‐sensitive tissues, thereby facilitating glucose uptake and improving glucose homeostasis [16]. Furthermore, galanin signalling has been associated with modulation of insulin resistance, inflammatory responses, appetite control, and lipid metabolism [17].

Although growing evidence supports the involvement of spexin and galanin in metabolic regulation and T2DM pathophysiology, no comprehensive quantitative synthesis has systematically evaluated circulating levels of these neuropeptides in patients with T2DM. Therefore, the present systematic review and meta‐analysis aimed to comprehensively assess circulating spexin and galanin concentrations in patients with T2DM by synthesising evidence from available observational studies.

## 2 Methods

This systematic review and meta‐analysis was conducted in accordance with the Preferred Reporting Items for Systematic Reviews and Meta‐Analyses (PRISMA 2020) guidelines [18]. The protocol of this review was prospectively registered in PROSPERO (ID: CRD420261308637).

### 2.1 Data Sources and Search Strategy

MEDLINE/PubMed, Scopus and Web of Science were systematically searched for studies reporting circulating spexin and galanin in type 2 diabetes mellitus. The electronic search was done using the following key terms: Spexin, SPX, neuropeptide Q, NPQ, Galanin, type 2 diabetes mellitus, T2DM, T2D, type 2 diabetes, diabetes type 2, diabetes mellitus type 2. The literature search was limited to studies published up to March 22, 2026, and the bibliographies of relevant articles were also hand‐searched. The search was limited to studies conducted on humans and English language publications. The complete search strategy for all databases is explained in Table S1.

### 2.2 Eligibility Criteria

#### 2.2.1 Inclusion Criteria

The inclusion criteria were developed according to the PECOS (Population, Exposure, Comparator, Outcomes, and Study design) framework. Studies were included if they: (1) involved adult participants diagnosed with Type 2 Diabetes Mellitus (Population); (2) assessed circulating levels of Spexin and/or Galanin in serum or plasma (Exposure); (3) included healthy or non‐diabetic individuals as comparator groups (Comparator); (4) reported quantitative outcomes related to circulating Spexin and/or Galanin concentrations, with sufficient data for effect size estimation (Outcomes); and (5) were observational studies, including cross‐sectional or case–control studies published in peer‐reviewed journals (Study design).

#### 2.2.2 Exclusion Criteria

The following studies were excluded:

1. Reviews, editorials, conference abstracts, case reports, letters, and animal studies.
2. Studies involving type 1 diabetes mellitus, gestational diabetes, or paediatric populations.
3. Studies lacking an appropriate control group.
4. Studies with insufficient data for quantitative synthesis.

### 2.3 Study Selection

All retrieved studies were imported into Rayyan. Ai for reference management and removal of duplicate records. Two reviewers (RKM and SY) independently screened the titles, abstracts, and full‐text articles to assess study eligibility. Any disagreements arising during the selection process were resolved through discussion and consensus before inclusion of the studies in the final analysis.

### 2.4 Data Extraction

Two independent investigators (RKM and SY) performed the initial data extraction. These data included first authors' names, year of publication, country, study design, study subjects, diagnostic criteria of T2DM, parameters measured, measurement method of spexin and galanin, mean spexin and galanin levels with corresponding standard deviations, type of biological sample (serum or plasma), and other relevant information. Numerical values were extracted using WebPlotDigitizer (version 5.2) in articles where data were presented graphically. A third investigator (KN) checked the accuracy of the data.

### 2.5 Risk of Bias

The risk of bias for each included case–control study was evaluated using the Newcastle‐Ottawa Quality Assessment Scale (NOS) [19]. Studies were appraised across four key domains: (1) selection of the study population, (2) ascertainment of exposure, (3) comparability of groups, and (4) ascertainment of outcome. A maximum score of 9 points was attainable for each study. Studies scoring ≤ 4 points were categorised as low quality (high risk of bias), 5–6 points as moderate quality (moderate risk of bias), and ≥ 7 points as high quality (low risk of bias). The risk of bias for each included cross‐sectional study was evaluated using the Joanna Briggs Institute (JBI) Critical Appraisal Checklist for Analytical Cross‐Sectional Studies [20]. Studies exhibiting ≤ 49% “yes” responses were classified as having a high risk of bias (low quality), those with 50%–69% as moderate risk of bias (moderate quality), and those with ≥ 70% as low risk of bias (high quality). The assessment of risk of bias was conducted independently by two reviewers (RKM and SY). Discrepancies were resolved through discussion and consensus among all reviewers.

### 2.6 Certainty of Evidence

The certainty of evidence for each outcome was assessed using the Grading of Recommendations Assessment, Development and Evaluation (GRADE) approach [21, 22]. As all included studies were observational in design, the certainty of evidence was initially rated as low. The certainty was subsequently downgraded, where appropriate, based on the domains of risk of bias, inconsistency, indirectness, imprecision, and publication bias. The evidence was also evaluated for potential upgrading based on a large magnitude of effect, a dose–response gradient, and whether all plausible residual confounding would reduce the observed effect; however, none of the outcomes met the criteria for upgrading. The overall certainty of evidence for each outcome was categorised as high, moderate, low, or very low.

### 2.7 Statistical Analysis

Data were analysed using R software (version 4.4.1) with the “meta” and “metasens” packages. Medians with ranges or interquartile ranges were converted to means and standard deviations using validated methods [23, 24, 25]. A random‐effects model was used for pooled analyses due to anticipated methodological and clinical variability across the studies. Continuous outcomes were summarised as standardised mean differences (SMDs) with 95% confidence intervals (CIs). Heterogeneity was assessed using Cochran's Q statistic and quantified by the I^2^ metric. Substantial heterogeneity was defined as a Q‐test *p*‐value < 0.10 and I^2^ > 50%. Prediction intervals were calculated to estimate the range of true effects expected in future studies. Forest plots and drapery plots were generated to visually present the pooled effect estimates. Sensitivity analyses were performed to evaluate the robustness of the findings. Subgroup analyses were performed based on study sample size (< 100 vs. ≥ 100 participants), specimen type (serum vs. plasma), study design (cross‐sectional vs. case–control), geographic region (Asian vs. transcontinental studies) and enzyme‐linked immunosorbent assay (ELISA) kit manufacturer origin (China‐based vs. India‐based vs. USA‐based). Studies evaluating diabetic nephropathy, diabetic peripheral neuropathy, diabetic retinopathy, and cardiovascular disease were grouped together as “T2DM with complications” because of the limited number of studies evaluating individual diabetic complications. Publication bias was assessed visually with funnel plots and statistically with Egger's regression test and Begg's rank correlation test. Doi plots were generated, with asymmetry interpreted by the Luis Furuya–Kanamori (LFK) index [26]. The trim‐and‐fill method was employed to estimate the impact of potentially missing studies and to derive adjusted pooled estimates. Meta‐regression was conducted to investigate the effects of mean age, mean BMI, mean HbA1c, proportion of male participants, and publication year on the pooled effect size and between‐study heterogeneity. Statistical significance was set at a two‐sided *p*‐value < 0.05, unless otherwise stated.

## 3 Results

### 3.1 Characteristics of Included Studies

**FIGURE 1:**
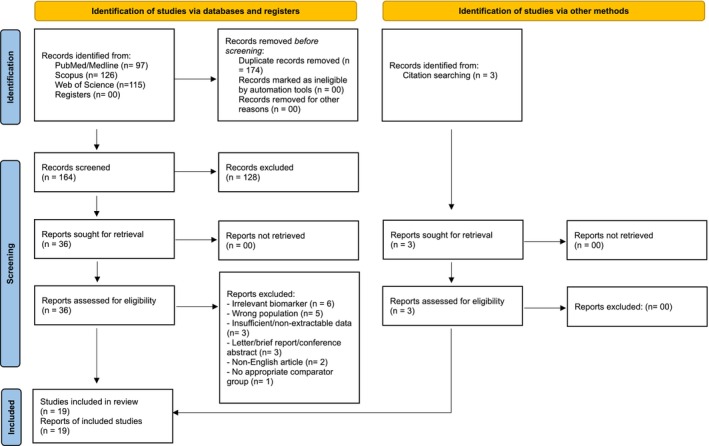
PRISMA flow diagram for the study selection process.

Initially, 338 records were identified through electronic database searching, comprising PubMed/MEDLINE (*n* = 97), Scopus (*n* = 126), and Web of Science (*n* = 115). Following the removal of 174 duplicate records, 164 unique records were retained for title and abstract screening. Of these, 128 records were excluded due to irrelevance to the study objective. The full texts of the remaining 36 reports were subsequently assessed for eligibility; all were successfully retrieved. During full‐text evaluation, 20 reports were excluded for various reasons, including irrelevant biomarkers (*n* = 6), wrong population (*n* = 5), insufficient or non‐extractable data (*n* = 3), letter/brief report/conference abstract (*n* = 3), non‐English articles (*n* = 2), and absence of an appropriate comparator group (*n* = 1). Additionally, three studies were identified through citation searching. Consequently, a total of 19 studies [5, 8, 9, 10, 11, 12, 13, 27, 28, 29, 30, 31, 32, 33, 34, 35, 36, 37, 38] met the eligibility criteria and were included in the systematic review and meta‐analysis (Figure 1). The key characteristics as well as study quality of the included studies are summarised in Table 1. The included studies were published between 2015 and 2025 and were conducted across diverse geographical regions, including China [8, 11, 30, 32], India [13, 31, 34], Iraq [27, 28, 35, 36, 37], Iran [10, 29], Turkey [12, 33, 38], Egypt [5], and Kuwait [9]. Among the included studies, 11 employed a cross‐sectional design [5, 8, 9, 10, 11, 13, 29, 30, 31, 33, 38], whereas the remaining 8 were case–control studies [12, 27, 28, 32, 34, 35, 36, 37]. Serum was the most frequently utilised biological sample, although a few studies assessed plasma spexin levels. Studies quantified circulating spexin and galanin levels using ELISA methods, and detailed information regarding assay kits is presented in Table S2.

**TABLE 1:** Baseline characteristics of studies included in meta‐analysis.

Author	Year	Country	Study design	Study subjects	Diagnostic criteria of type 2 DM	Measured parameter	Sample	Quality	Main findings
Abdel Salam MS et al. [5]	2025	Egypt	Cross‐sectional study	90 participants (30 T2DM, 35 FDRs, 25 healthy controls); only T2DM vs. control groups were included in the meta‐analysis	ADA	Spexin	Serum	High	Serum spexin levels were significantly lower in T2DM patients compared to controls, with a progressive decline observed from controls to FDRs to T2DM, indicating its role in insulin resistance and metabolic dysfunction.
Abed BA et al. [27]	2021	Iraq	Case–control study	120 subjects (30 controls, 30 T2DM, 30 T2DM + hypothyroidism, 30 T2DM + hyperthyroidism); only T2DM vs. control groups were included in the meta‐analysis	Previously diagnosed T2DM (criteria not clearly specified)	Galanin	Serum	High	Galanin levels were significantly higher in T2DM and further elevated in T2DM with thyroid dysfunction. Galanin showed a strong association with insulin resistance and metabolic parameters, suggesting its role as a biomarker in T2DM and thyroid‐related metabolic disturbances.
Aboutorabi R et al. [10]	2025	Iran	Cross‐sectional study	97 T2DM patients (33 with diabetic nephropathy and 64 without nephropathy)	ADA	Spexin	Serum	High	Serum spexin levels were significantly lower in T2DM patients with diabetic nephropathy compared with those without nephropathy.
Al‐fatlawiy HDA et al. [28]	2022	Iraq	Case–control	60 T2DM patients vs. 30 healthy controls	Not reported	Spexin	Serum	High	Serum spexin levels were significantly decreased in T2DM patients compared to controls. Spexin showed a negative correlations with insulin resistance (HOMA‐IR), HbA1c, BMI, and lipid parameters, and positive correlation with HDL‐C and HOMA‐β, suggesting its role as a potential biomarker for insulin resistance and glycemic control.
Amirpour M et al. [29]	2021	Iran	Cross‐sectional study	168 participants (42 normal healthy, 42 obese healthy, 42 normal‐weight T2DM, 42 obese T2DM)	Not reported	Spexin	Serum	High	No significant difference in spexin levels between diabetic and non‐diabetic groups. However, spexin showed an inverse correlations with fasting glucose, triglycerides, and waist circumference in non‐diabetic individuals, suggesting its role in metabolic regulation rather than direct association with T2DM.
Celik F et al. [12]	2022	Turkey	Case–control study	120 participants (30 T2DM + cataract, 30 DRP + cataract, 30 cataract only, 30 healthy controls); cataract only patients were not included in the meta‐analysis.	ADA criteria	Spexin	Plasma	High	Blood spexin levels were significantly lower in T2DM patients with cataract compared to healthy controls and showed an inverse association with glucose and HbA1c, suggesting a role in diabetic metabolic dysregulation and complications.
Dai J et al. [30]	2023	China	Cross‐sectional study	462 participants (52 healthy controls, 109 FDRs, 115 IGR, 80 newly diagnosed T2DM, 106 established T2DM); only T2DM vs. control groups were included in the meta‐analysis.	WHO	Spexin	Serum	High	Serum spexin levels varied across the glycemic continuum, showing a bell‐shaped pattern: Increased in prediabetes and decreased in established T2DM. Spexin was negatively correlated with FPG, HbA1c, and insulin resistance, and positively associated with β‐cell function.
Gowdu T. et al. [31]	2021	India	Cross‐sectional study	330 subjects (110 controls, 110 T2DM, 110 T2DM + HTN)	ADA	Spexin	Serum	High	Spexin levels were significantly decreased in T2DM and further reduced in T2DM + HTN. Spexin showed an inverse association with glycemic indices, insulin resistance, liver enzymes, and inflammation, indicating its role as a negative biomarker in metabolic dysfunction.
Gu L et al. (1) [32]	2015	China	Case–control study	121 T2DM patients and 105 healthy controls	Not reported	Spexin	Serum	Moderate	Serum spexin levels were significantly lower in T2DM compared to controls. Spexin showed a strong negative correlations with FBG, HbA1c, TG, and LDL‐C, indicating its role in glucose and lipid metabolism.
Gu L et al. (2) [8]	2022	China	Cross‐sectional study	364 participants (41 NGT controls, 323 newly diagnosed T2DM [89 obese, 161 overweight, 73 normal‐weight])	WHO	Spexin	Serum	High	Serum spexin levels were significantly lower in T2DM patients compared to controls and varied according to BMI, with the lowest levels observed in obese individuals. Spexin showed a strong negative associations with BMI, visceral fat, and insulin resistance, and increased following improvement in metabolic status.
Karaca A et al. [33]	2018	Turkey	Cross‐sectional study	82 participants (29 T1DM, 30 T2DM, 23 healthy controls); only T2DM vs. control groups were included in the meta‐analysis.	ADA	Spexin	Serum	High	Spexin levels were significantly lower in both T1DM and T2DM compared to controls. No significant correlation between spexin and glycemic parameters, lipids, or BMI was observed, suggesting spexin reduction may be independent of metabolic control.
Khadir A et al. [9]	2020	Kuwait	Cross‐sectional study	185 participants (50 normal‐weight controls, 66 obese without T2DM, 69 obese with T2DM); only obese with T2DM vs. control groups were included in the meta‐analysis.	Not reported	Spexin	Plasma	High	Spexin levels were significantly reduced in obese individuals with and without T2DM compared to normal‐weight controls. No significant difference between obese T2DM and obese non‐diabetic groups, indicating that spexin reduction is more strongly associated with obesity than diabetes.
Liu Y et al. [11]	2024	China	Cross‐sectional study	167 T2DM patients (56 without DPN, 67 painless DPN, and 44 painful DPN)	WHO	Spexin	Serum	High	Serum spexin concentrations progressively decreased from non‐DPN to painless DPN and were lowest in painful DPN.
Mishra RK et al. [34]	2025	India	Case–control study	100 participants (50 T2DM patients, 50 healthy controls)	International Diabetes Federation (IDF) criteria	Galanin	Serum	High	Serum galanin levels were significantly higher in T2DM patients compared to healthy controls and showed a strong positive associations with fasting glucose, insulin resistance (HOMA‐IR), BMI, and triglycerides, indicating its role in metabolic dysregulation and potential as a predictive biomarker of T2DM.
Russell SA et al. [35]	2023	Iraq	Case–control study	42 type 2 diabetes mellitus and 42 healthy controls	Diagnosed Based on fasting serum glucose testing after at least 8 h of fasting; however, the specific diagnostic criteria were not reported.	Galanin	Serum	High	Serum galanin levels were significantly elevated in patients with T2DM compared to healthy controls and showed a strong positive association with fasting serum glucose.
Shamkhi FH et al. [36]	2025	Iraq	Case–control study	200 participants (100 T2DM women, 100 healthy controls)	WHO & ADA criteria	Spexin	Serum	High	Serum spexin levels were significantly lower in T2DM patients compared to controls.
Shareef MS et al. [37]	2023	Iraq	Case–control study	180 postmenopausal women (90 newly diagnosed T2DM, 90 non‐diabetic controls)	Newly diagnosed T2DM (physician diagnosed); diagnostic criteria not reported	Spexin	Serum	High	Spexin levels were significantly reduced in T2DM patients compared to controls. Spexin showed a negative association with glycemic parameters and insulin resistance, suggesting its role as a biomarker in T2DM pathophysiology and metabolic dysfunction.
Tejaswi G et al. [13]	2021	India	Cross‐sectional study	330 participants (110 controls, 110 T2DM, 110 T2DM + CVD)	ADA	Spexin	Serum	High	Serum spexin levels were significantly decreased in T2DM patients compared to controls and further reduced in T2DM with CVD, indicating its role as a potential biomarker for insulin resistance and cardiovascular risk in T2DM.
Yilmaz U et al. [38]	2024	Türkiye	Cross‐sectional study	154 participants (49 T2DM + ED, 55 ED without T2DM, 50 healthy controls); only T2DM + ED vs. control groups were included in the meta‐analysis.	ESC/EASD guidelines for T2DM	Galanin	Serum	High	Serum galanin levels were significantly higher in individuals with T2DM and erectile dysfunction compared to controls, while lower levels were observed in ED without T2DM, indicating a complex interaction between metabolic and reproductive factors.

Abbreviations: ADA, American Diabetes Association; BMI, body mass index; CVD, cardiovascular disease; DM, diabetes mellitus; DPN, diabetic peripheral neuropathy; DRP, diabetic retinopathy; ED, erectile dysfunction; ESC/EASD, European Society of Cardiology/European Association for the Study of Diabetes; FBG, fasting blood glucose; FDRs, first‐degree relatives; FPG, fasting plasma glucose; FSG, fasting serum glucose; HbA1c, glycated haemoglobin; HDL‐C, high‐density lipoprotein cholesterol; HOMA‐β, homeostatic model assessment of β‐cell function; HOMA‐IR, homeostatic model assessment of insulin resistance; HTN, hypertension; IDF, International Diabetes Federation; IGR, impaired glucose regulation; LDL‐C, low‐density lipoprotein cholesterol; NGT, normal glucose tolerance; T1DM, type 1 diabetes mellitus; T2DM, type 2 diabetes mellitus; TG, triglycerides; WHO, World Health Organization. Only study groups relevant to the meta‐analysis comparison were included in the pooled analysis where applicable. FDRs, obese non‐diabetic participants, cataract‐only participants, ED without T2DM participants, and other non‐eligible comparator groups were excluded from quantitative synthesis.

### 3.2 Spexin in T2DM

#### 3.2.1 Meta‐Analysis and Sensitivity Analysis

**FIGURE 2:**
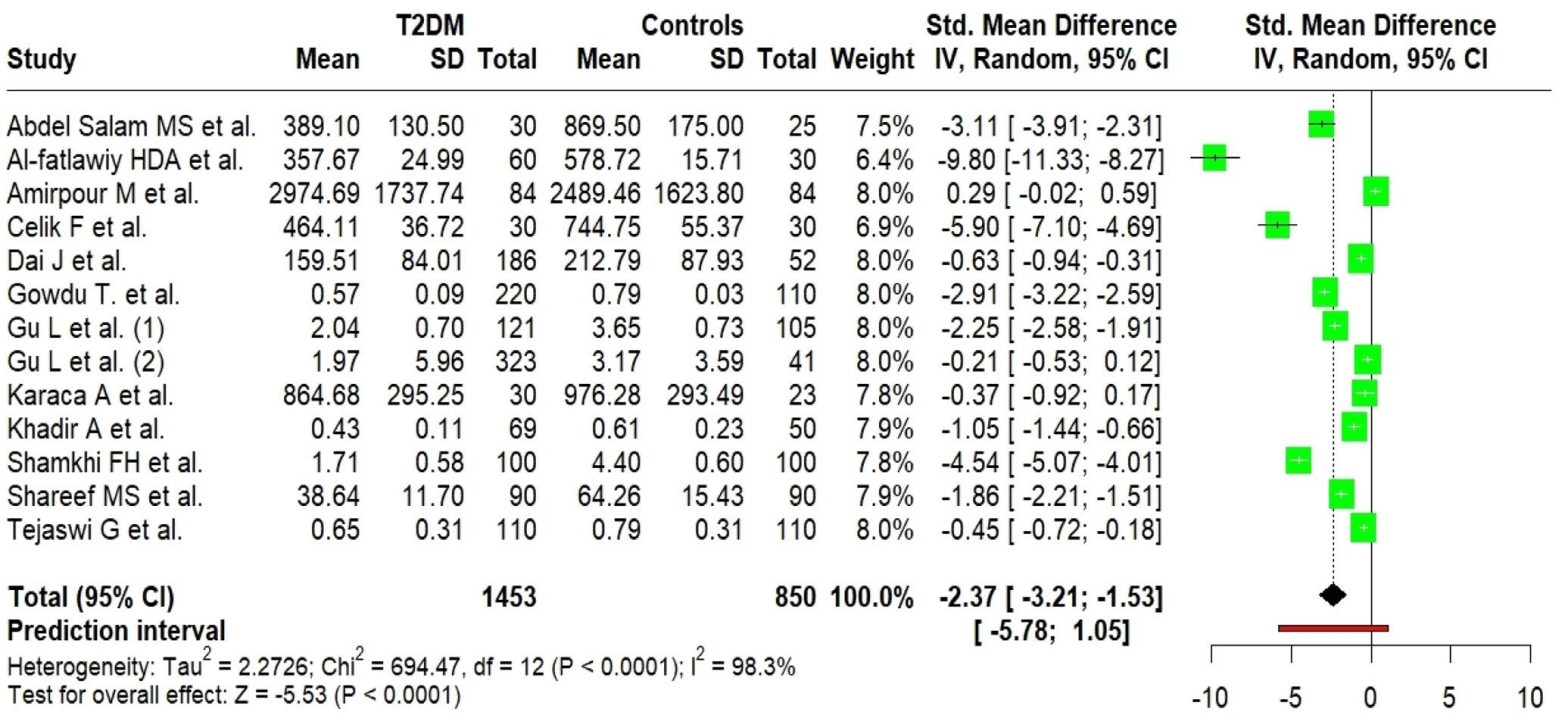
Forest plot showing comparison of circulating spexin levels between T2DM and controls.

**FIGURE 3:**
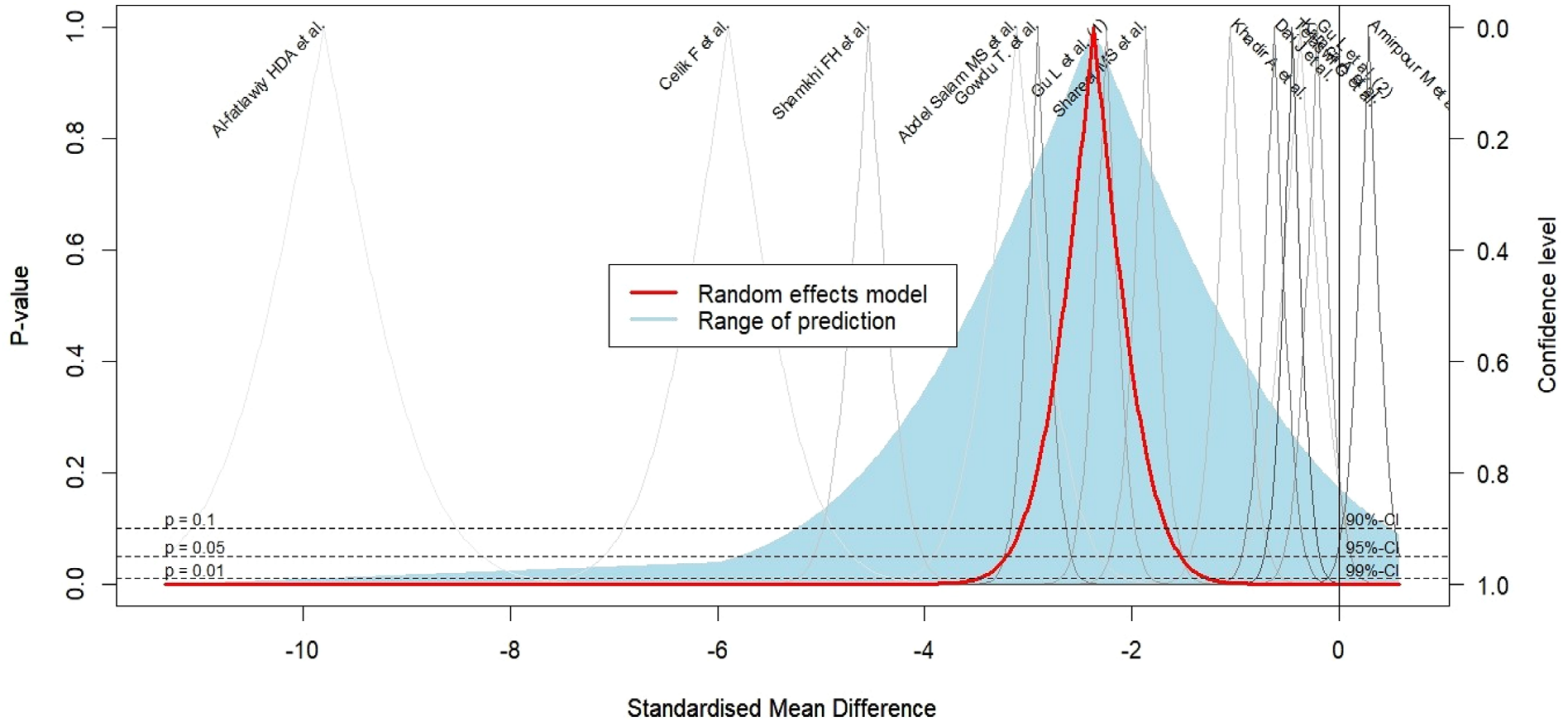
Drapery plot depicting the meta‐analysis of spexin results based on study‐specific *p*‐value functions in T2DM.

A total of 13 studies [5, 8, 9, 12, 13, 28, 29, 30, 31, 32, 33, 36, 37], encompassing 1453 patients diagnosed with T2DM and 850 controls, were incorporated into the quantitative synthesis assessing circulating spexin concentrations. The pooled analysis, employing a random‐effects model, revealed that individuals with T2DM exhibited significantly lower circulating spexin levels in comparison to non‐diabetic controls (SMD = −2.37, 95% CI: −3.21 to −1.53; *p* < 0.0001). Substantial between‐study heterogeneity was observed (I^2^ = 98.3%, τ^2^ = 2.2726, *p* < 0.0001). The prediction interval ranged from −5.78 to 1.05, indicating substantial variability in both the magnitude and direction of effects across future populations (Figure 2). This variability suggests that contextual or methodological factors may partially influence the observed association. A drapery plot is presented in Figure 3 to visualise the meta‐analysis results, based on the *p*‐value functions of each study. Sensitivity analysis employing a sequential leave‐one‐out methodology revealed consistently robust findings. The exclusion of any individual study did not significantly affect the pooled effect estimate, with pooled SMDs consistently remaining statistically significant, ranging from −1.85 to −2.59. Furthermore, heterogeneity persisted at elevated levels across all iterations (I^2^ ranging from 98.0% to 98.4%), suggesting that no single study exerted a disproportionate influence on the overall results (Figure S1).

#### 3.2.2 Subgroup Analyses

Stratification by sample size showed significantly lower spexin levels in both smaller (< 100 participants) and larger (≥ 100 participants) studies. The pooled effect was more pronounced in smaller studies (SMD = −4.74, 95% CI: −8.25 to −1.23) than in larger studies (SMD = −1.50, 95% CI: −2.37 to −0.63), but the subgroup difference was not significant (*p* = 0.0792). Subgroup analysis by biological sample type indicated that serum samples showed significantly lower spexin concentrations in T2DM patients (SMD = −2.21, 95% CI: −3.12 to −1.29; *p* < 0.0001), while plasma samples showed a non‐significant association (SMD = −3.44, 95% CI: −8.19 to 1.31; *p* = 0.1563). No significant difference was found between serum‐ and plasma‐based studies (*p* = 0.6181). When stratified by study design, both cross‐sectional and case–control studies revealed lower circulating spexin levels in T2DM patients. However, the magnitude of association varied significantly: Cross‐sectional studies produced an SMD of −1.03 (95% CI: −1.82 to −0.24), whereas case–control studies exhibited a stronger association, with an SMD of −4.70 (95% CI: −6.33 to −3.06). The subgroup difference was statistically significant (*p* < 0.0001), suggesting study design contributed to heterogeneity. Subgroup analysis by geographic region revealed consistently lower circulating spexin levels among T2DM patients in both Asian and transcontinental populations. Transcontinental populations showed a significant reduction (SMD = −3.09, 95% CI: −6.06 to −0.11; *p* = 0.0418, I^2^ = 97.5%), as did Asian populations (SMD = −2.18, 95% CI: −3.10 to −1.25; *p* < 0.0001, I^2^ = 98.5%). However, the test for subgroup differences was not significant (*p* = 0.5660). Subgroup analysis by ELISA kit manufacturer origin showed consistently diminished circulating spexin levels in T2DM patients. China‐based kits showed a significant reduction (SMD = −2.93, 95% CI: −4.31 to −1.55; *p* < 0.0001, I^2^ = 98.3%), as did India‐based kits (SMD = −1.74, 95% CI: −3.22 to −0.25; *p* = 0.0220, I^2^ = 98.5%). USA‐based kits indicated a non‐significant association (SMD = −1.92, 95% CI: −4.20 to 0.35; *p* = 0.0973, I^2^ = 98.9%). The test for subgroup differences was non‐significant (χ^2^ = 1.45, *p* = 0.4846), suggesting assay manufacturer origin did not significantly moderate the pooled effect. Detailed subgroup analyses are presented in Table 2. Results are visualised in the supplemental materials (Figure S2−S6).

**TABLE 2:** Subgroup analysis of circulating spexin levels in patients with T2DM.

Subgroup variable	Subgroup	Studies (k)	T2DM (n)	Controls (n)	Pooled SMD (95% CI)	p‐vale	I^2^ (%)	τ^2^	Test for Subgroup Difference
Sample size	< 100	4	150	108	−4.74 (−8.25 to −1.23)	0.0081	98.3	12.5202	χ^2^ = 3.08, *p* = 0.0792
	≥ 100	9	1303	742	−1.50 (−2.37 to −0.63)	0.0007	98.4	1.7365	
Sample type	Serum	11	1354	770	−2.21 (−3.12 to −1.29)	< 0.0001	98.4	2.3111	χ^2^ = 0.25, *p* = 0.6181
	Plasma	2	99	80	−3.44 (−8.19 to 1.31)	0.1563	98.2	11.5498	
Study design	Cross‐sectional	8	1052	495	−1.03 (−1.82 to −0.24)	0.0102	97.4	1.2380	χ^2^ = 15.66, *p* < 0.0001
	Case–control	5	401	355	−4.70 (−6.33 to −3.06)	< 0.0001	97.8	3.2752	
Geographical region	Asian	10	1363	772	−2.18 (−3.10 to −1.25)	< 0.0001	98.5	2.1582	χ^2^ = 0.33, *p* = 0.5660
	Transcontinental	3	90	78	−3.09 (−6.06 to −0.11)	0.0418	97.5	6.6960	
ELISA kit origin	China‐based	7	541	349	−2.93 (−4.31 to −1.55)	< 0.0001	98.3	3.2949	χ^2^ = 1.45, *p* = 0.4846
	India‐based	3	420	310	−1.74 (−3.22 to −0.25)	0.0220	98.5	1.6999	
	USA‐based	3	492	191	−1.92 (−4.20 to 0.35)	0.0973	98.9	3.9917	

Abbreviations: SMD: standardised mean difference; CI: confidence interval; T2DM: type 2 diabetes mellitus; τ^2^: between‐study variance.

#### 3.2.3 Publication Bias

**FIGURE 4:**
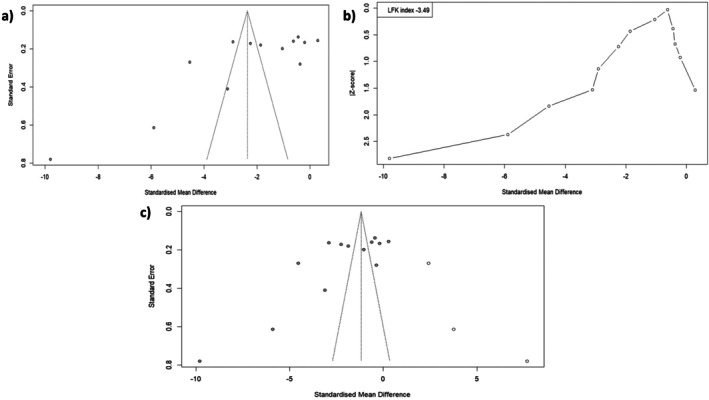
(a) Funnel plot showing publication bias; (b) Doi plot showing publication bias; (c) Trim‐and‐fill funnel plot. Three missing studies were imputed on the right side. The imputed studies are represented by circles that have no fill colour.

The assessment of publication bias revealed significant asymmetry. Visual inspection of the funnel plot indicated pronounced small‐study effects and an asymmetrical distribution of studies surrounding the pooled effect size (Figure 4a). This observation was further corroborated by the Doi plot, which exhibited considerable asymmetry with an LFK index of −3.49, indicative of substantial publication bias (Figure 4b). Additionally, Egger's regression test demonstrated significant funnel plot asymmetry (*p* = 0.0218), while Begg and Mazumdar's rank correlation test also suggested potential small‐study effects (*p* = 0.0281). Trim‐and‐fill analysis identified three potentially missing studies (Figure 4c). After adjustment, the pooled effect size remained statistically significant (adjusted SMD = −1.18, 95% CI: −2.09 to −0.27; *p* = 0.0107), indicating that the observed decrease in circulating spexin levels in T2DM remained robust despite the potential influence of publication bias.

#### 3.2.4 Meta‐Regression

Only mean age significantly moderated the pooled effect size (QM = 7.27, *p* = 0.007), showing an inverse relationship with effect size (β = −0.1586, 95% CI: −0.2739 to −0.0433) and accounting for 16.97% of heterogeneity. Mean BMI, HbA1c, proportion of male participants, and publication year were not significant moderators. Despite moderator inclusion, substantial residual heterogeneity remained across all models (residual I^2^ 97.79%–98.41%; all QE *p* < 0.0001), suggesting other unmeasured factors contribute to variability (Table 3). In addition, meta‐regression bubble plots assessing the influence of mean age, mean BMI, mean HbA1c, proportion of male participants, and publication year on the SMD in circulating spexin levels are presented in Figure S7−S11.

**TABLE 3:** Meta‐regression analyses exploring sources of heterogeneity.

Moderator	k	β‐Coefficient	SE	95% CI	QM	*p*‐value	R^2^ (%)	τ^2^	I^2^ (%)
Mean age	13	−0.1586	0.0588	−0.2739 to −0.0433	7.2713	0.0070	16.97	1.8869	97.95
Mean BMI	12	−0.0770	0.1489	−0.3688 to 0.2148	0.2672	0.6052	0.00	2.5897	98.28
Mean HbA1c	11	−0.5104	0.5342	−1.5575 to 0.5367	0.9128	0.3394	0.00	1.8735	97.79
Male sex (%)	11	−0.0087	0.0205	−0.0488 to 0.0314	0.1804	0.6710	5.85	2.4173	98.03
Publication year	13	−0.2531	0.1724	−0.5910 to 0.0847	2.1563	0.1420	0.00	2.5215	98.41

Abbreviations: β, regression coefficient; SE, standard error; CI, confidence interval; QM, test of moderators; R^2^, proportion of between‐study heterogeneity explained by the moderator; τ^2^, residual between‐study variance; I^2^, residual heterogeneity; k, number of studies included in the meta‐regression.

### 3.3 Spexin in Patients With Diabetes Complications

A pooled random‐effects meta‐analysis of four studies [10, 11, 12, 13] revealed significantly lower circulating spexin concentrations in patients with diabetic complications (T2DM + C) relative to those without complications (T2DM − C) (SMD = −1.12, 95% CI: −1.75 to −0.49; *p* = 0.0005). Substantial between‐study heterogeneity was observed (I^2^ = 90.3%, τ^2^ = 0.3660, *p* < 0.0001), indicative of considerable variability across the included studies. Furthermore, the prediction interval, ranging from −3.30 to 1.06, indicated that although the pooled effect was consistent with reduced spexin levels in patients with complications, the magnitude and direction of the association could vary across future populations and clinical settings (Figure S12). A drapery plot depicting the meta‐analysis results based on study‐specific *p*‐value functions is shown in Figure S13. Sensitivity analysis, employing a leave‐one‐out approach, confirmed the overall robustness of the pooled findings. Sequential exclusion of individual studies did not materially alter the direction or statistical significance of the association, with pooled SMDs consistently ranging from −0.73 to −1.31. Specifically, the exclusion of the study by Aboutorabi et al. [10] substantially reduced heterogeneity to 0.0%. In contrast, the omission of the remaining studies continued to exhibit persistently high heterogeneity (I^2^ ranging from 91.5% to 93.5%). Despite this observed variability, all sensitivity analyses maintained statistical significance, thereby confirming that no single study disproportionately influenced the overall pooled estimate (Figure S14). The included studies encompassed a range of diabetic complications, specifically diabetic retinopathy (DR), diabetic peripheral neuropathy (DPN), diabetic nephropathy (DN), and cardiovascular disease (CVD). Notwithstanding the heterogeneity in complication phenotypes, all investigations consistently reported reduced circulating spexin levels in patients with complicated T2DM relative to those with uncomplicated T2DM.

### 3.4 Galanin in T2DM

**FIGURE 5:**
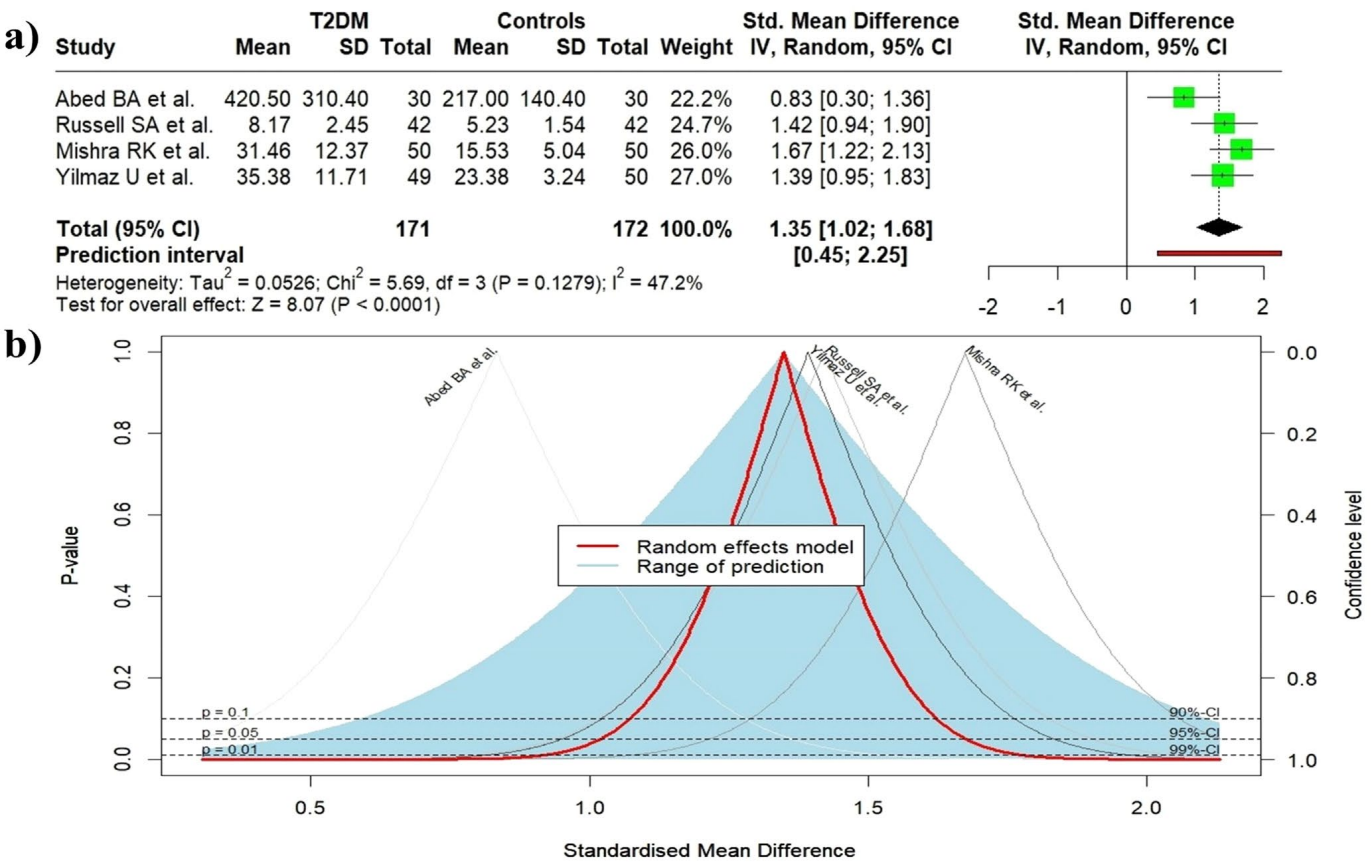
(a) Forest plot showing comparison of circulating galanin levels between T2DM and controls. (b) Drapery plot depicting the meta‐analysis of circulating galanin levels in patients with T2DM.

Four studies [27, 34, 35, 38], encompassing 171 patients with T2DM and 172 healthy controls, were incorporated into a meta‐analysis evaluating circulating galanin levels. The pooled analysis demonstrated that circulating galanin levels were significantly elevated in patients with T2DM compared with healthy controls, with a pooled standardised mean difference (SMD) of 1.35 (95% CI: 1.02–1.68; *p* < 0.0001), indicative of a large effect size. Moderate heterogeneity was observed among the included studies (I^2^ = 47.2%, *p* = 0.1279), implying a reasonable degree of inter‐study consistency. Furthermore, the prediction interval, ranging from 0.45 to 2.25, indicated that the direction of effect is likely to remain positive in future studies despite observed between‐study variability (Figure 5a). Drapery plot of circulating galanin levels in T2DM, showing study‐specific *p*‐value functions, is shown in Figure 5b. Sensitivity analysis, employing a leave‐one‐out approach, substantiated the robustness of the findings. Sequential exclusion of individual studies did not materially alter the pooled effect estimate; the pooled SMDs consistently ranged from 1.24 to 1.50, with all subsequent analyses retaining statistical significance (*p* < 0.0001). Specifically, omission of the study by Abed BA et al. [27] eliminated heterogeneity (0.0%), while the exclusion of other individual studies yielded moderate heterogeneity, ranging from 38.7% to 64.7%. Collectively, these results indicate that no single study exerted undue influence on the aggregate pooled estimate, thereby corroborating the stability and reliability of the observed association between elevated circulating galanin levels and T2DM (Figure S15). A publication bias analysis for galanin was not conducted due to the limited number of included studies (*n* = 4) in the meta‐analysis.

### 3.5 Diagnostic Accuracy of Spexin and Galanin

Diagnostic accuracy data were reported in only six of the included studies [5, 10, 13, 34, 35, 37]. Among studies evaluating spexin, Abdel Salam et al. [5] reported that serum spexin effectively discriminated patients with T2DM from a non‐diabetic population with an Area Under the Curve (AUC) of 0.92, using a cut‐off value of ≤ 485 ng/L, with 90.0% sensitivity and 85.7% specificity. The comparator group, however, comprised both healthy controls and first‐degree relatives of patients with T2DM. Shareef et al. [37] revealed an AUC of 0.909, with a cut‐off value of 46.33 pg/mL, sensitivity of 85.6%, and specificity of 77.8% for discriminating newly diagnosed T2DM from healthy postmenopausal women. Tejaswi et al. [13] reported a modest diagnostic performance for T2DM vs. healthy controls (AUC 0.65, cut‐off 0.62 ng/mL, 65% sensitivity, 62% specificity), whereas diagnostic performance was higher for identifying T2DM with cardiovascular disease (AUC 0.83, cut‐off 0.51 ng/mL, 81% sensitivity, 62% specificity). Aboutorabi et al. [10] evaluated serum spexin for identifying diabetic nephropathy among patients with T2DM and reported an AUC of 0.986 with a cut‐off value of 81.10 ng/mL, yielding 95.3% sensitivity and 94.1% specificity.

Among studies evaluating galanin, Mishra et al. [34] reported excellent diagnostic performance with an AUC of 0.920, an optimal cut‐off value of > 19.135 pg/mL, and sensitivity and specificity rates of 86.0% and 78.0%, respectively, for distinguishing patients with T2DM from healthy controls. Russell et al. [35] similarly demonstrated good diagnostic performance, reporting an AUC of 0.858, a cut‐off value of > 6.14 ng/mL, and sensitivity and specificity rates of 76.74% each for discriminating between T2DM patients and healthy controls.

### 3.6 Certainty of Evidence

The certainty of evidence for all three outcomes was assessed using the GRADE approach. Overall, the certainty of evidence was rated as very low for all outcomes (Table S3). For circulating spexin levels in patients with T2DM vs. controls, the certainty was downgraded because of very serious inconsistency and strongly suspected publication bias. The certainty of evidence for circulating spexin levels in patients with diabetic complications vs. those without complications was downgraded because of very serious inconsistency and serious imprecision. The certainty of evidence for circulating galanin levels in patients with T2DM vs. controls was downgraded because of serious imprecision. None of the outcomes met the GRADE criteria for upgrading; therefore, no upgrading of the certainty of evidence was applied.

## 4 Discussion

The present systematic review and meta‐analysis provides comprehensive evidence demonstrating significant alterations in circulating spexin and galanin concentrations among patients with T2DM. The pooled findings revealed markedly reduced circulating spexin concentrations in individuals with T2DM compared with non‐diabetic controls, whereas galanin concentrations were significantly elevated. Collectively, these findings corroborate the emerging paradigm that neuropeptides implicated in energy homeostasis and metabolic regulation may exert pivotal roles in the pathophysiology of T2DM and its associated metabolic disturbances.

Spexin, a highly conserved neuropeptide composed of 14 amino acids, has garnered significant attention due to its multifaceted metabolic functions. In addition to its role in appetite regulation and energy expenditure, spexin plays a crucial part in maintaining glucose and lipid metabolism, while also exerting modulatory effects on cardiovascular physiology. Recent studies have implicated spexin in the development and progression of obesity, insulin resistance, and T2DM. The co‐localisation of spexin with insulin in pancreatic β‐cells further underscores its potential involvement in pancreatic function and endocrine regulation, suggesting that alterations in spexin signalling may significantly influence glucose homeostasis and overall metabolic health [39].

A significant finding of the present meta‐analysis is the consistently lower circulating spexin concentrations observed in individuals with T2DM compared to healthy control subjects. This observation is biologically plausible and is corroborated by an expanding body of experimental and clinical evidence that implicates spexin in the regulation of glucose homeostasis, insulin sensitivity, lipid metabolism, appetite control, and overall energy balance. Gu et al. [32] reported significantly reduced circulating spexin levels in patients with T2DM, demonstrating inverse associations with fasting plasma glucose, HbA1c, triglycerides, and low‐density lipoprotein cholesterol (LDL‐C), thereby suggesting a critical role for spexin in metabolic regulation. Similarly, Karaca et al. [33] identified markedly diminished serum spexin concentrations in both Type 1 and Type 2 diabetes, indicating that alterations in spexin signalling may represent a broader metabolic disturbance associated with diabetic conditions. The inverse association between spexin and markers of insulin resistance, as identified in several studies, further substantiates this hypothesis. Abdel Salama et al. [5] demonstrated significant negative correlations between spexin and BMI, fasting insulin, Homeostatic Model Assessment of Insulin Resistance (HOMA‐IR), triglycerides, and total cholesterol. These findings suggest that decreasing levels of spexin may be associated with deteriorating metabolic function, potentially occurring prior to the onset of overt diabetes. Notably, Dai et al. [30] reported a bell‐shaped distribution of spexin across the glycemic continuum, revealing elevated levels in first‐degree relatives with impaired glucose regulation, followed by a marked decline in individuals with established T2DM. This pattern may indicate an initial compensatory upregulation of spexin in response to early metabolic stress, which is subsequently followed by exhaustion or dysregulation during chronic hyperglycemia and progressive β‐cell dysfunction. Such findings are particularly compelling, as they imply that spexin may function not merely as a passive biomarker but potentially as an adaptive metabolic peptide involved in the maintenance of glucose homeostasis. Genetic evidence further substantiates the role of spexin in the susceptibility to diabetes. Shamkhi et al. [36] identified significant associations between two polymorphisms of the SPX gene, rs761986956 and rs1214680179, and circulating spexin concentrations in Iraqi women. Specifically, the GG genotype of rs761986956 and the CC genotype of rs1214680179 were correlated with lower serum spexin levels and an increased likelihood of T2DM. Although the precise biological mechanisms underlying these associations remain to be elucidated, these genetic variants may impact gene transcription, protein stability, or the binding of regulatory factors. Collectively, these findings suggest that spexin may represent a potential biomarker of metabolic dysfunction and a possible therapeutic target; however, these implications require confirmation in high‐quality prospective and mechanistic studies. Whether interventions designed to restore or enhance spexin signalling can be translated into effective strategies for the prevention and management of T2DM remains to be determined.

Several included studies reported inverse associations between circulating spexin levels and measures of adiposity and metabolic dysfunction, including BMI, waist circumference, visceral fat, and dyslipidemia. Importantly, Khadir et al. [9] and Gu et al. [8] demonstrated that improvements in metabolic health through exercise and therapeutic interventions were accompanied by increases in spexin concentrations, suggesting that spexin may serve as a dynamic biomarker reflecting metabolic improvement and treatment responsiveness.

Another important finding of this meta‐analysis is the observed association between diminished spexin levels and the development of diabetic complications, thereby suggesting that spexin dysregulation extends beyond its established role in glucose metabolism to encompass vascular, renal, neural, and inflammatory pathways. Specifically, Celik et al. [12] identified reduced spexin concentrations in both the serum and aqueous humour of diabetic patients presenting with retinopathy and cataract, indicating a potential link between spexin deficiency and ocular microvascular complications. Tejaswi et al. [13] further reported a decline in circulating spexin levels among individuals with T2DM complicated by cardiovascular disease, which implies that progressive metabolic and vascular dysfunction may exacerbate impairments within the spexinergic system. Renal complications also demonstrate spexin involvement. Aboutorabi et al. [10] observed lower spexin levels in patients with diabetic nephropathy, thereby supporting its potential renoprotective function. Experimental evidence from Cha et al. [40] corroborated this by demonstrating that spexin‐based activation of galanin receptors attenuated diabetic nephropathy in murine models through the suppression of renal fibrotic pathways, amelioration of histopathological injury, and reduction of albuminuria. Furthermore, Yazgan et al. [41] and Memi et al. [42] have highlighted spexin's modulatory role in inflammatory and fibrotic responses in chronic kidney disease, suggesting that spexin may confer protection against progressive renal damage. Spexin is additionally implicated in diabetic peripheral neuropathy (DPN). Liu et al. [11] reported an inverse association between serum spexin levels and the odds of developing DPN. Beyond this clinical correlation, the authors investigated spexin's mechanistic role in neuropathic progression and pain modulation. Given that chronic low‐grade inflammation contributes significantly to DPN pathogenesis, experimental studies elucidating spexin's potent anti‐inflammatory effects are particularly relevant. Spexin administration has been shown to reduce circulating interleukin‐6 (IL‐6) in high‐fructose diet‐induced metabolic syndrome models and to inhibit macrophage recruitment in obese mice [43, 44]. Liu et al. [11] also demonstrated that spexin treatment suppressed lipopolysaccharide‐induced expression of pro‐inflammatory cytokines (IL‐6, IL‐1β, TNF‐α) in human peripheral blood mononuclear cells. These collective findings suggest that diminished spexin levels in DPN patients may compromise anti‐inflammatory defences, thereby facilitating neuroinflammation and neural injury. Collectively, these observations position spexin as a pleiotropic metabolic and anti‐inflammatory peptide with potential protective effects against a spectrum of diabetic complications. The consistent reduction of circulating spexin observed across nephropathy, neuropathy, retinopathy, and cardiovascular disease suggests that spexin deficiency may reflect a shared pathophysiological pathway linking chronic hyperglycemia, inflammation, oxidative stress, endothelial dysfunction, and tissue fibrosis in T2DM.

Galanin, another key neuropeptide evaluated in the present meta‐analysis, has been suggested to play significant roles in the neuroendocrine axis for the mediation of diverse neuronal pathological conditions of ageing, feeding and energy metabolism, pancreatic secretion of insulin and diabetes, mood and behaviour, seizure, pain experience, bone density, cartilage growth plate physiology and repair and skin [45]. Our pooled findings showing altered circulating galanin concentrations in T2DM are consistent with emerging clinical evidence. Mishra et al. [34] demonstrated significantly elevated serum galanin levels among patients with T2DM compared with healthy controls in a North Indian population, with strong positive correlations observed between galanin and BMI, triglycerides, fasting insulin, HOMA‐IR, fasting glucose, and HbA1c. Similarly, Abed et al. [27] reported significantly higher galanin concentrations in patients with T2DM, particularly among those with concurrent thyroid dysfunction, suggesting that galanin may serve as a predictor of insulin resistance and metabolic dysregulation. The elevated circulating galanin levels observed in patients with T2DM may represent a compensatory neuroendocrine response to chronic insulin resistance and impaired glucose utilisation. Experimental studies have demonstrated that galanin enhances insulin sensitivity, promotes GLUT4 expression and translocation, and facilitates glucose uptake in peripheral tissues [16, 46]. However, despite these beneficial metabolic effects, circulating galanin concentrations are consistently increased in individuals with T2DM. Fang et al. [47] proposed the concept of “galanin resistance” to explain this paradox, suggesting that chronic obesity, hyperglycemia, and insulin resistance induce compensatory galanin hypersecretion while diminished galanin receptor activity and impaired downstream signalling blunt its biological efficacy. Consequently, elevated galanin levels may reflect an adaptive attempt to restore glucose homeostasis in the presence of reduced galanin responsiveness, analogous to the development of hyperinsulinemia during insulin resistance.

Despite these important findings, several limitations warrant acknowledgement. Considerable heterogeneity was observed across included studies, potentially reflecting variations in population characteristics, ethnicity, glycemic status, obesity burden, disease duration, medication status, and assay methodologies. The predominant cross‐sectional or case–control designs of the included studies inherently limit the ability to infer causality. Furthermore, variations in ELISA kits and laboratory protocols may have contributed to inter‐study variability in measured neuropeptide concentrations. Certain subgroup analyses were constrained by a limited number of available studies, particularly concerning galanin. Moreover, the potential for publication bias could not be entirely excluded. The subgroup analysis pertaining to “T2DM with complications” necessitates careful interpretation due to the inherent heterogeneity of included complications, such as diabetic nephropathy, peripheral neuropathy, diabetic retinopathy, and cardiovascular disease, which exhibit distinct underlying pathophysiological mechanisms and varying degrees of severity.

Nevertheless, the present meta‐analysis possesses several important strengths. To our knowledge, this represents one of the most comprehensive syntheses evaluating both circulating spexin and galanin in T2DM. The study incorporated data from multiple geographic regions and included a substantial number of participants. Furthermore, the use of random‐effects modelling, subgroup analyses, sensitivity analyses, prediction intervals, and drapery plots enhanced the robustness and interpretability of the findings.

Future research should focus on conducting well‐designed, adequately powered prospective diagnostic accuracy studies to validate the clinical utility of spexin and galanin as diagnostic biomarkers for T2DM. These studies should utilise standardised assay methods and include well‐defined comparator groups to enhance comparability across studies. Furthermore, multicentre studies involving ethnically diverse populations are needed to externally validate the diagnostic performance of these biomarkers. Longitudinal studies are also warranted to determine whether alterations in circulating spexin and galanin precede the onset of T2DM and its complications and to evaluate their potential roles in early diagnosis, risk stratification, and disease monitoring.

## 5 Conclusion

In conclusion, the present meta‐analysis demonstrates that circulating spexin levels are significantly decreased, whereas galanin levels are significantly elevated, in patients with T2DM, including those with diabetic complications. These findings suggest that both neuropeptides may be involved in the metabolic disturbances underlying T2DM and its complications. However, these findings should be interpreted with caution given the very low certainty of the available evidence. Further prospective studies, including well‐designed diagnostic accuracy and mechanistic investigations, are warranted to validate these findings and clarify the clinical utility of spexin and galanin in T2DM and its complications.

## Author Contributions

**Amrendra P. Kushwaha:** methodology, data curation, investigation, formal analysis, supervision, writing – review and editing, writing – original draft, project administration, visualization, validation. **Nand Kishor Prasad Sah:** methodology, formal analysis, investigation, writing – review and editing. **Roshan Kumar Mahat:** conceptualization, methodology, software, data curation, formal analysis, validation, investigation, visualization, project administration, resources, writing – original draft, writing – review and editing, supervision. **Sushil Yadav:** methodology, data curation, investigation, formal analysis, writing – review and editing. **Kahkashan Naaz:** methodology, data curation, formal analysis, validation, investigation, writing – review and editing.

## Funding

The authors have nothing to report.

## Ethics Statement

The authors have nothing to report.

## Consent

The authors have nothing to report.

## Conflicts of Interest

The authors declare no conflicts of interest.

## Supporting information


**Table S1:** Search strategies in databases.
**Table S2:** Details of ELISA kits used for the measurement of Spexin and Galanin.
**Table S3:** GRADE evidence profile for all reported outcomes.
**Figure S1:** Leave‐one‐out sensitivity analysis of circulating spexin levels in patients with type 2 diabetes mellitus (T2DM).
**Figure S2:** Subgroup analysis of circulating spexin levels in patients with type 2 diabetes mellitus according to study sample size.
**Figure S3:** Subgroup analysis of circulating spexin levels in patients with type 2 diabetes mellitus according to biological sample type.
**Figure S4:** Subgroup analysis of circulating spexin levels in patients with type 2 diabetes mellitus according to study design.
**Figure S5:** Subgroup analysis of circulating spexin levels in patients with type 2 diabetes mellitus according to geographic region.
**Figure S6:** Subgroup analysis of circulating spexin levels in patients with type 2 diabetes mellitus according to ELISA kit manufacturer origin.
**Figure S7:** Meta‐regression bubble plot assessing the influence of mean age on the standardised mean difference (SMD) in circulating spexin levels between patients with type 2 diabetes mellitus and healthy controls.
**Figure S8:** Meta‐regression bubble plot assessing the influence of mean BMI on the standardised mean difference (SMD) in circulating spexin levels between patients with type 2 diabetes mellitus and healthy controls.
**Figure S9:** Meta‐regression bubble plot assessing the influence of mean HbA1c on the standardised mean difference (SMD) in circulating spexin levels between patients with type 2 diabetes mellitus and healthy controls.
**Figure S10:** Meta‐regression bubble plot assessing the influence of male % on the standardised mean difference (SMD) in circulating spexin levels between patients with type 2 diabetes mellitus and healthy controls.
**Figure S11:** Meta‐regression bubble plot assessing the influence of publication year on the standardised mean difference (SMD) in circulating spexin levels between patients with type 2 diabetes mellitus and healthy controls.
**Figure S12:** Spexin levels between T2DM with complications (T2DM + C) and without complications (T2DM‐C).
**Figure S13:** Drapery plot depicting the meta‐analysis results based on study‐specific *p*‐value functions in T2DM with complications (T2DM + C).
**Figure S14:** Sensitivity analysis illustrating the influence of individual studies on the pooled estimate of spexin levels for T2DM with complications.
**Figure S15:** Sensitivity analysis illustrating the influence of individual studies on the pooled estimate of galanin levels in T2DM.

## Data Availability

The data that supports the findings of this study are available in the Supporting Information of this article.
