# Supplementary material for: Neuropeptide Alterations in Type 2 Diabetes Mellitus: A Meta‐Analysis of Circulating Spexin and Galanin Levels
Source: Endocrinol Diabetes Metab. 2026 Aug 10;9(5):e70303. doi: 10.1002/edm2.70303 (PMC13455544; doi:10.1002/edm2.70303)
Supplement: Supplementary file 1 [file EDM2-9-e70303-s001.docx]

**Neuropeptide Alterations in Type 2 Diabetes Mellitus: A Meta-Analysis of Circulating Spexin and Galanin Levels**

**Table S1**: Search strategies in databases.

| **Databases** | **Search terms** | **No of articles identified** |
| --- | --- | --- |
| PubMed/Medline | ("Spexin"[All Fields] OR "SPX"[All Fields] OR "neuropeptide Q"[All Fields] OR "NPQ"[All Fields] OR "Galanin"[All Fields]) AND ("type 2 diabetes mellitus"[All Fields] OR "T2DM"[All Fields] OR "T2D"[All Fields] OR "type 2 diabetes"[All Fields] OR "diabetes type 2"[All Fields] OR "diabetes mellitus type 2"[All Fields]) | 97 |
| Scopus | TITLE-ABS-KEY ( ( "Spexin" OR "SPX" OR "neuropeptide Q" OR "NPQ" OR "Galanin" ) AND ( "type 2 diabetes mellitus" OR "T2DM" OR "T2D" OR "type 2 diabetes" OR "diabetes type 2" OR "diabetes mellitus type 2" ) ) | 126 |
| Web of Science | (“Spexin” OR “SPX” OR “neuropeptide Q” OR “NPQ” OR “Galanin”) AND (“type 2 diabetes mellitus” OR “T2DM” OR “T2D” OR “type 2 diabetes” OR “diabetes type 2” OR “diabetes mellitus type 2”) | 115 |

**Table S2:** Details of ELISA kits used for the measurement of Spexin and Galanin.

| **Authors** | **ELISA kit used** |
| --- | --- |
| Abdel Salam MS et al. | Bioassay Technology Lab, Shanghai, China |
| Abed BA et al. | Al-Shkairate establishments, Jordan |
| Aboutorabi R et al. | Shanghai Crystal Day Biotech Co., China. |
| Al-fatlawiy HDA et al. | MLSIN kit, China |
| Amirpour M et al. | Shanghai Crystal Day Biotech Co., China |
| Celik F et al. | Bioassay Technology Laboratory, Shanghai, China |
| Dai J et al. | Fine biotech co., Wuhan, Hubei, China |
| Gowdu T. et al. | Krishgen biosys­tems, India |
| Gu L et al. (1) | CUSABIO, Wuhan, China |
| Gu L et al. (2) | My BioSource, USA |
| Karaca A et al. | SunRed Biotechnology Co., Shanghai, China |
| Khadir A et al. | Phoenix Pharmaceuticals, Burlingame, California, USA |
| Liu Y et al. | Fine Biotech Co., Wuhan, Hubei, China |
| Mishra RK et al. | KRISHGEN Biosystems, INDIA |
| Russell SA et al. | Elabscience Inc, USA |
| Shamkhi FH et al. | ABclonal, USA |
| Shareef MS et al. | Krishgen Biosystems India |
| Tejaswi G et al. | Krishgen biosystems India |
| Yilmaz U et al. | SunRed Biotechnology, China |

**Table S3:** GRADE evidence profile for all reported outcomes.

| Outcome | No. of studies | Risk of bias | Inconsistency | Indirectness | Impression | Publication bias | Other considerations* | Overall certainty of evidence |
| --- | --- | --- | --- | --- | --- | --- | --- | --- |
| Circulating spexin in T2DM vs controls | 13 | Not serious | Very serious^a^ | Not serious | Not serious | Strongly suspected^c^ | None | Very low  ⊕◯◯◯ |
| Spexin in T2DM with complications vs without complications | 4 | Not serious | Very serious^a^ | Not serious | Serious^b^ | Not assessed | None | Very low  ⊕◯◯◯ |
| Circulating galanin in T2DM vs controls | 4 | Not serious | Not serious | Not serious | Serious^b^ | Not assessed | None | Very low  ⊕◯◯◯ |

*Large effect, plausible confounding, and dose response gradient; ^a^Downgraded two levels for very serious inconsistency due to substantial unexplained heterogeneity, wide variability in effect estimates, and prediction intervals crossing the null effect; ^b^Downgraded one level for serious imprecision because the estimate was based on only four studies with limited sample size; ^c^Downgraded one level because publication bias was strongly suspected based on funnel plot asymmetry, significant Egger's and Begg's tests, major Doi plot asymmetry. Publication bias was not downgraded for the galanin and complications analyses because fewer than 10 studies were available, precluding reliable assessment.

**Figure S1:** Leave-one-out sensitivity analysis of circulating spexin levels in patients with type 2 diabetes mellitus (T2DM).

**Figure S2:** Subgroup analysis of circulating spexin levels in patients with type 2 diabetes mellitus according to study sample size.

**Figure S3:** Subgroup analysis of circulating spexin levels in patients with type 2 diabetes mellitus according to biological sample type.

**Figure S4:** Subgroup analysis of circulating spexin levels in patients with type 2 diabetes mellitus according to study design.

**Figure S5:** Subgroup analysis of circulating spexin levels in patients with type 2 diabetes mellitus according to geographic region.

**Figure S6:** Subgroup analysis of circulating spexin levels in patients with type 2 diabetes mellitus according to ELISA kit manufacturer origin.

**Figure S7:** Meta-regression bubble plot assessing the influence of mean age on the standardized mean difference (SMD) in circulating spexin levels between patients with type 2 diabetes mellitus and healthy controls.

**Figure S8:** Meta-regression bubble plot assessing the influence of mean BMI on the standardized mean difference (SMD) in circulating spexin levels between patients with type 2 diabetes mellitus and healthy controls.

**Figure S9:** Meta-regression bubble plot assessing the influence of mean HbA1c on the standardized mean difference (SMD) in circulating spexin levels between patients with type 2 diabetes mellitus and healthy controls.

**Figure S10:** Meta-regression bubble plot assessing the influence of male % on the standardized mean difference (SMD) in circulating spexin levels between patients with type 2 diabetes mellitus and healthy controls.

**Figure S11:** Meta-regression bubble plot assessing the influence of publication year on the standardized mean difference (SMD) in circulating spexin levels between patients with type 2 diabetes mellitus and healthy controls.

**Figure S12:** Spexin levels between T2DM with complications (T2DM+C) and without complications (T2DM-C).

**Figure S13:** Drapery plot depicting the meta-analysis results based on study-specific p-value functions in T2DM with complications (T2DM+C).

**Figure S14:** Sensitivity analysis illustrating the influence of individual studies on the pooled estimate of spexin levels for T2DM with complications.

**Figure S15:** Sensitivity analysis illustrating the influence of individual studies on the pooled estimate of galanin levels in T2DM.
